# Differences in the Expression of Insulin‐Like Growth Factor Signaling Pathway Members in Patients With Psoriasis Vulgaris and Controls

**DOI:** 10.1155/mi/2136373

**Published:** 2026-02-02

**Authors:** Holmannova Drahomira, Borska Lenka, Fiala Zdenek, Krejsek Jan, Hamakova Kvetoslava, Cermakova Eva, Rehacek Vit, Fiala Ondrej, Maresova Tereza, Borsky Pavel

**Affiliations:** ^1^ Department of Preventive Medicine, Faculty of Medicine in Hradec Kralove, Charles University, Hradec Kralove, 500 03, Czech Republic, cuni.cz; ^2^ Department of Clinical Immunology and Allergology, University Hospital and Faculty of Medicine in Hradec Kralove, Charles University, Hradec Kralove, 500 03, Czech Republic, cuni.cz; ^3^ Clinic of Dermal and Venereal Diseases, University Hospital Hradec Kralove, Hradec Kralove, 500 03, Czech Republic, fnhk.cz; ^4^ Department of Medical Biophysics, Faculty of Medicine in Hradec Kralove, Charles University, Hradec Kralove, 50003, Czech Republic, cuni.cz; ^5^ Transfusion Center, University Hospital Hradec Kralove, Hradec Kralove, 500 03, Czech Republic, fnhk.cz

**Keywords:** IGF, IGF-1, IGF-2, psoriasis

## Abstract

Insulin‐like growth factors (IGFs) and IGF–binding proteins (IGFBPs) regulate cell proliferation, differentiation, metabolic processes, and immune activities. Psoriasis is a systemic inflammatory disease with metabolic disorders as an important comorbidity in the pathogenesis of which members of the IGF family could also play a role. Therefore, we decided to evaluate the levels of members of the IGF signaling pathway in patients with psoriasis. Sixty‐nine people were enrolled in our study: 34 patients with psoriasis and 35 controls. The following parameters were evaluated in serum obtained from peripheral blood: total cholesterol, triglycerides, high‐density lipoprotein, fasting glucose, IGF‐1, IGF‐1R, IGF‐2, IGF‐2R, IGFBP1, IGFBP2, IGFBP3, IGFBP4, IGFBP6, and insulin. The levels of several parameters differed between groups. The levels of fasting glucose, insulin, IGFBP3, and IGFBP6 were higher in patients with psoriasis, while the levels of IGF‐1, IGF‐1R, and IGBP4 were higher in controls. The results suggested that the IGF‐1 signaling pathway can be involved in the pathogenesis of psoriasis and its comorbidities, especially metabolic disorders such as insulin resistance, diabetes, and metabolic syndrome. The novelty of our study is in its comprehensive assessment of the involvement of the IGF‐1 signaling pathway in the pathogenesis of psoriasis and advances the understanding of the pathogenesis of psoriasis and its comorbidities.

## 1. Introduction

Psoriasis is a chronic systemic inflammatory disease that affects ~3% of the world’s population [1]. It is a multifactorial disease, with both internal and external factors involved in its pathogenesis. Among internal factors, genetic predisposition, immune system dysfunction, and skin dysbiosis play a significant role [2]. Triggers that activate the pathological immune response include environmental, chemical, physical exposures (environmental pollution, irritation by chemicals, mechanical pressure, and UV radiation), infections, hormonal changes, diet, and the presence of other diseases, e.g., diabetes, metabolic syndrome, and autoimmune diseases [3–5]. Furthermore, psoriasis can potentiate the development and progression of these ones, including neurodegenerative diseases and cancer [6, 7].

The main changes in immune system reactivity that are associated with psoriasis are the activation of resident immune cells and induction of activation and migration of peripheral immune cells into the skin lesion. Both innate and acquired immunity are activated. Functionally polarized Th17 T cell subset plays an important role in the pathogenesis of psoriasis. Increased immune cell activity is associated with an increase in the production of proinflammatory cytokines TNF‐α, IL‐6, and IL‐17; growth factors vascular endothelial growth factor (VEGF), transforming growth factor β (TGF‐β), epidermal growth factor (EGF); oxidative stress; and increase risk of DNA damage. The proinflammatory microenvironment in the skin promotes abnormal proliferation and differentiation of keratinocytes and enhances angiogenesis [8, 9]. Psoriasis presents externally through distinctive skin manifestations marked by inflammation, redness, and the appearance of flaking silvery scales. These lesions predominantly appear on the extensor surfaces of the elbows and knees. Additionally, psoriasis can affect the nails and joints, potentially leading to psoriatic arthritis [10]. The condition often coexists with various comorbidities.

**Figure 1:**
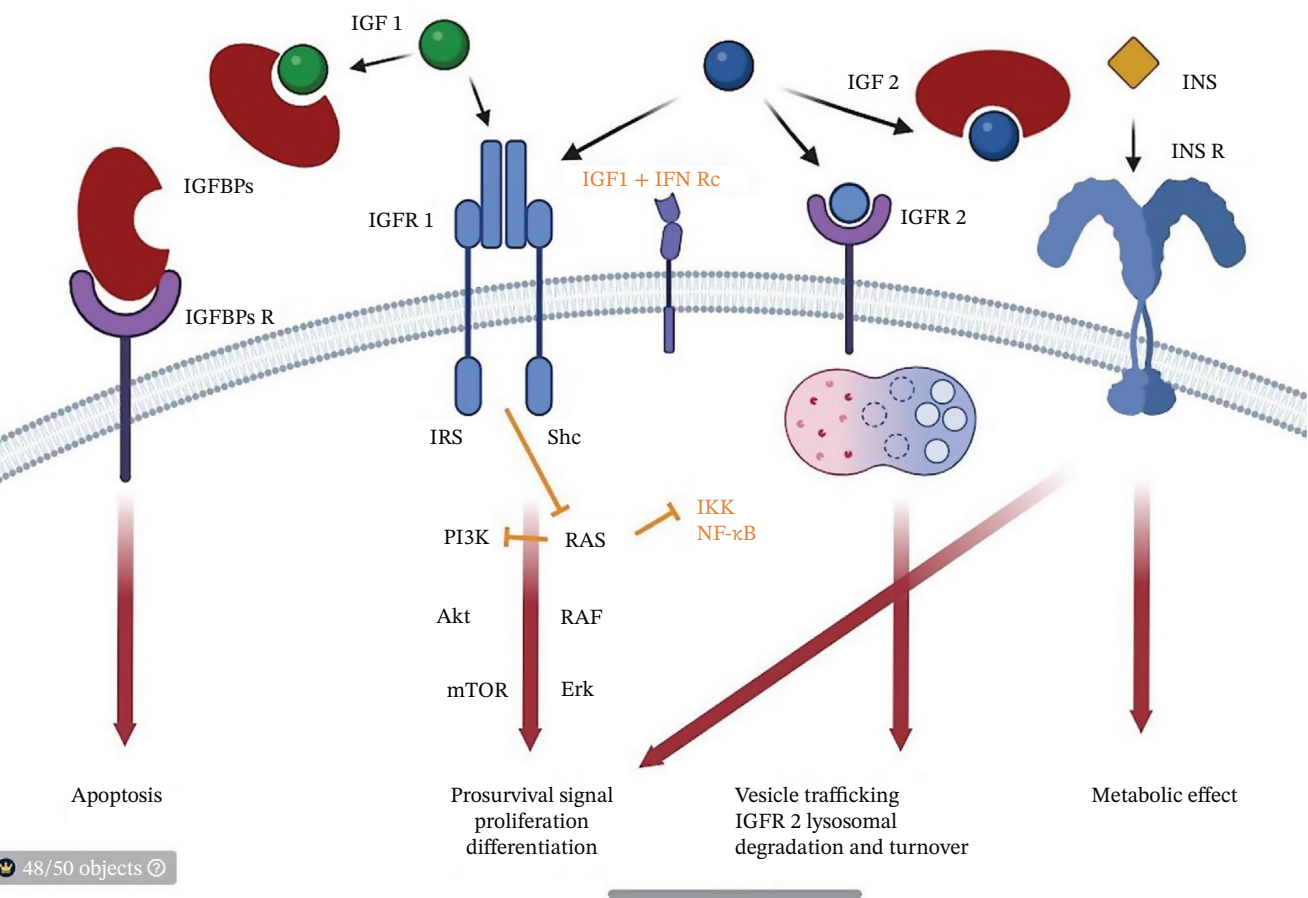
Schematic representation of IGF signaling network. Legend: IGF‐1 interacts with IGFR 1 and induce intracellular signaling pathway, including PI3K (phosphatidyl inositol 3 kinase), Akt (protein kinase B), mTOR (mammalian target of rapamycin) or/and RAS, RAF, and Erk (extracellular signal‐related kinase) which leads to cells survival, proliferation and differentiation, and IGBPs that limits its functions via reduced bioavailability. IGF‐2 binds IGFR 1 and IGFR 2 and influences its own turnover. INS (insulin) binds its receptor INS R and induce cell survival, proliferation, and differentiation and has metabolic effect. IGFBPs interact with their receptors and can induce apoptosis. Under the proinflammatory stimulation (orange arrows), for example, by interferon γ, the IGF‐1 can suppress RAS and other proinflammatory pathways (IKK, inhibitory kappa B kinase; NF‐κB, transcription factor regulating proinflammatory cytokine expression) [12] (BioRender).

The role of growth factors in the pathogenesis of psoriasis is only partially known. The IGF signaling pathway is a complex signaling network that controls various cellular processes such as cell proliferation, differentiation, survival, and migration. This pathway is involved in normal development, growth, aging, or angiogenesis. However, it is also participating in the pathogenesis of multiple diseases such as cancer, diabetes, metabolic syndrome (regulate insulin resistance and lipid metabolism), or neurodegenerative diseases [11]. IGF signaling pathways consist of two ligands and their receptors: IGF‐1 (somatomedin C) and IGF‐2 (somatomedin A) and IGF‐1R (CD22; insulin growth factor 1 receptor), IGF‐2R (CD222; insulin growth factor 2 receptor), and IGF–binding proteins (IGFBPs) (Figure 1) [13].

Expression of members of the IGF signaling network is influenced by various factors, for example, IGF‐1 expression depends on the level of growth hormone (GH), thrombin, TNF‐α, and reactive oxygen species (ROS), while estrogen negatively regulates its production. The expression of IGF‐2 is also driven by nutrition status, and IGFBP synthesis depends on nutrient availability, proinflammatory cytokines, and ROS [14, 15]. IGFBPs bind and regulate IGF‐1 and IGF‐2 activity and their bioavailability [16]. The IGFBP family consists of six members from IGFBP1 to IGFBP6. They differ in expression pattern and affinities to insulin‐like growth factors (IGFs). Binding of IGFs to IGFBPs can enhance (stabilize IGF–receptor interaction) and reduce (sequester IGF from receptors) the effects of IGFs [17]. However, IGFBPs have numerous motifs and binding sites and can bind heparin, extracellular matrix components, surface proteoglycans, and proteolytic cleavage sites [14, 16, 18].

Members of the IGF signaling pathway have been reported to have both a proinflammatory and an anti‐inflammatory effect depending on the context and the target tissue [19–22]. IGF‐1 involvement in psoriasis is proven [23]. It supports the excessive proliferation and survival of keratinocytes and the infiltration of the skin with immune cells and the release of proinflammatory cytokines. The role of IGF‐2 in psoriasis is not well established. In the IGFBP group, some members have proinflammatory and anti‐inflammatory functions, which can be further modified by the microenvironment [24].

In this study, we focused on the IGF signaling pathway and markers of metabolic disease (fasting glucose and blood lipids), as IGF signaling pathways are also involved in metabolism, in psoriasis patients and compared the results with matched controls. We analyzed the levels of insulin, IGF‐1, IGF‐2, IGF‐1R, IGF‐2R, and IGFBP1, 2, 3, 4, and 6 and correlated them with these metabolic markers.

So far, mostly only a few members of the IGF signaling pathways have been evaluated in individual studies. The aim of the study was to provide the coverage of most members for a more comprehensive view of the involvement of IGF pathways in the pathogenesis of psoriasis. Deeper understanding of the mechanisms involved in the pathogenesis of psoriasis can provide more targeted treatment to prevent damage to the body from chronic inflammation and the development of comorbidities.

## 2. Results

### 2.1. Levels of Members of IGF‐1 Signaling Pathway

**Figure 2:**
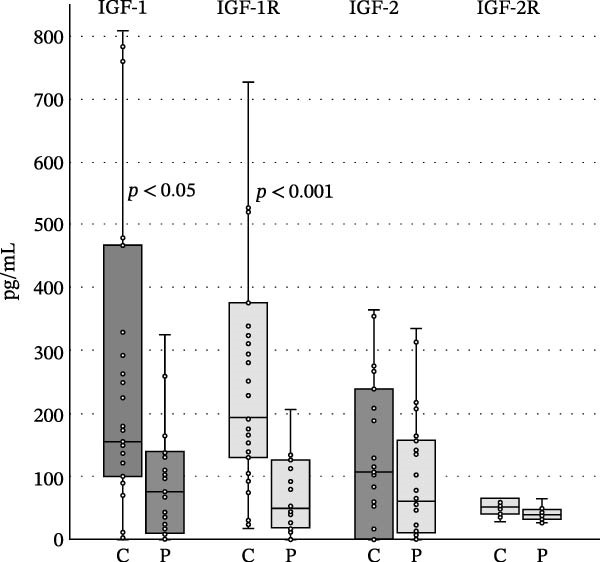
Differences between the levels of IGF‐1, IGF‐1R, IGF‐2, and IGF‐2R in controls and patients with psoriasis. Boundaries of the box represent 1^st^ and 3^rd^ quartile, a line within the box marks median. Whiskers above and below the box indicate the 10^th^ and 90^th^, and statistical difference is marked as a *p*‐value; C, controls, P, patients.

**Figure 3:**
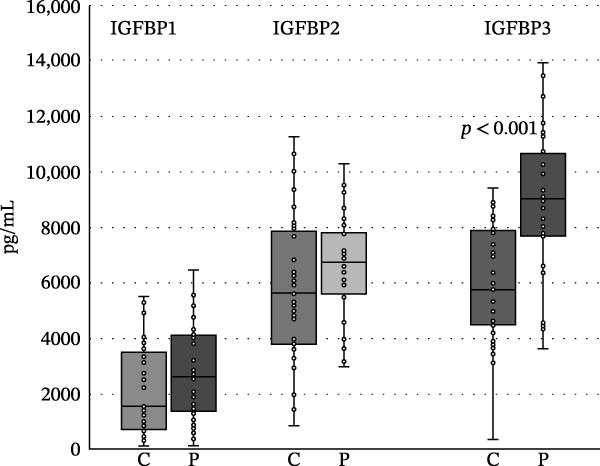
Differences between the levels of IGFBP1, IGFBP2, and IFGBP3 in controls and patients with psoriasis. Boundaries of the box represent 1^st^ and 3^rd^ quartile, a line within the box marks median. Whiskers above and below the box indicate the 10^th^ and 90^th^, and statistical difference is marked as a *p*‐value; C, controls, P, patients.

**Figure 4:**
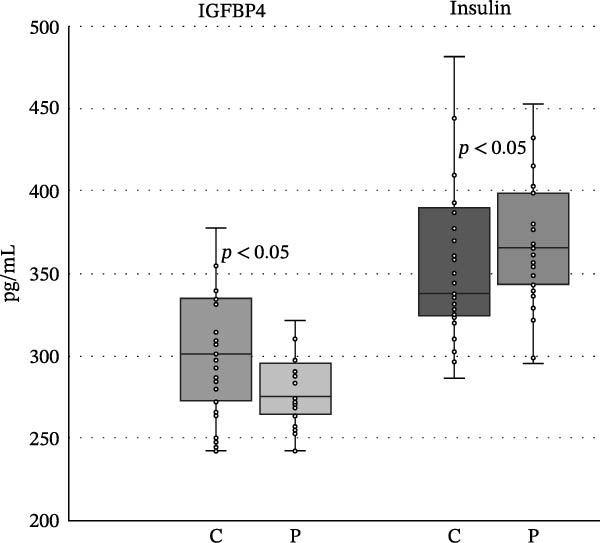
Differences between the levels of IGFBP4 and insulin in controls and patients with psoriasis. Boundaries of the box represent 1^st^ and 3^rd^ quartile, a line within the box marks median. Whiskers above and below the box indicate the 10^th^ and 90^th^, and statistical difference is marked as a *p*‐value; C, controls, P, patients.

**Figure 5:**
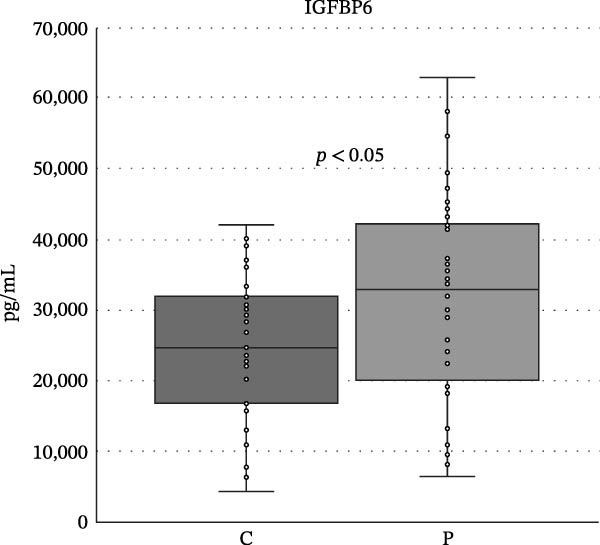
Differences between the levels of IGFBP6 in controls and patients with psoriasis. Boundaries of the box represent 1^st^ and 3^rd^ quartile, a line within the box marks median. Whiskers above and below the box indicate the 10^th^ and 90^th^, and statistical difference is marked as a *p*‐value; C, controls, P, patients.

We found no differences in the IGF‐2, IGF‐2R, IGFBP1, and IGFP2 values, but we did find statistically significant differences in IGF‐1, IGF‐R1, IGFBP3, IGFBP4, IGFBP6, and insulin levels between the patient and control groups (<0.05, <0.001, <0.001, <0.05, <0.05, <0.05, and <0.05). The levels of IGF‐1, IGF‐1R, and IGFBP4 were higher in the control group, while the levels of IGFBP1, IGFBP2, IGFBP3, IGFBP6, and insulin were increased in the patient group (Table 1 and Figures 2–5).

**Table 1:** Levels of analyzed members of IGF signaling pathway (pg/mL) in groups of controls and patients.

35/34	Q1	Q2	Q3	*p*‐value	35/34	Q1	Q2	Q3	*p*‐value
**IGF-1**					**IGFBP2**				

C	99.7	173.8	478.4	<0.05	C	3808.2	5642.9	7954.8	N
P	9.43	77.8	188.85		P	5494.25	6897.4	7934.85	

**IGF-1R**					**IGFBP3**				

C	105.3	194.3	376.5	<0.001	C	4474.2	5774.1	7934.6	<0.001
P	18.4	54.2	130.5		P	7404.4	9105.2	10,865.63	

**IGF-2**					**IGFBP4**				

C	0	104.95	246.23	N	C	273.1	300.8	335.1	<0.05
P	11.53	67.85	209.83		P	263.425	275.05	300.38	

**IGF-2R**					**IGFBP6**				

C	39.9	52.10	107.2	N	C	16,787	24,635.1	31,908.4	<0.05
P	33.0	40.95	67.2		P	19,372.68	32,853.95	42,433.82	

**IGFBP1**					**Insulin**				

C	688.9	1579.9	3648.0	N	C	324.1	337.8	392.4	<0.05
P	1334.7	2617.3	4186.48		P	343.3	365.2	398.58	

*Note:* Q1, Q2, and Q3, quartiles; C and P, controls and patients; N, nonsignificant difference.

### 2.2. Correlations Among Parameters

We detected several correlations among measured parameters in controls and patients with psoriasis. In both groups, the correlation between IGF‐2R and waist, total cholesterol, IGF‐1, and IGF‐1R was revealed. In the control group, IGF‐2R also correlated with insulin and IGFBP2, and in the patient group, IGF‐2R correlated with TAG. IGF‐1R correlated in both groups with IGF‐1 and previously mentioned IGF‐2R. Other interesting correlations are in the control group between HDL and IGFBP1 and IGF‐1 and insulin, in the patients group a negative correlation between TAG and IGFBP1 and a negative correlation between fasting glucose and IGFBP6 (Table 2). The strength of correlation between PASI and other parameters was very weak and weak (range 0.00–0.19 and 0.20–0.39). The strongest correlations were between PASI and BMI and PASI and waist circumference (0.31 and 0.34). The strength of correlation higher than 0.2 was between PASI and IGF‐1, IGFBP4, and IGFBP6 (0.22, −0.25, and 0.2). We also attempted multiple regression analysis. The model exhibited a low R‐squared value, indicating that the independent variables explain only a minimal percentage of the variability in the dependent variable (Supporting Information).

**Table 2:** Relationship among parameters in controls and patients with psoriasis.

Correlations controls (Spearman correlation)								
Parameter	Waist	HDL	CHOL	Insulin	IGF‐1	IGF‐1R	IGF‐2	IGFBP2
IGF‐2R	0.66	—	0.43	0.56	0.65	0.68	—	0.46
IGF‐1	—	—	—	0.63	—	0.60	0.63	—
IGFBP1	—	0.40	—	—	—	—	—	0.40

Correlations patients (Spearman correlation)								
	Waist	CHOL	TAG	GLU	IGF‐1	IGF‐1R	IGF‐2	IGFBP4
IGF‐2R	0.55	0.47	0.92	—	0.52	0.71	—	—
IGF‐1	—	—	—	—	—	0.82	0.55	0.42
IGFBP6	—	—	—	−0.56	—	—	—	—
IGFBP1	—	—	−0.48	—	—	—	—	—
Insulin	—	—	—	0.54	—	—	—	—

*Note:* Absolute value *r* (Spearman correlation); only medium, strong, or very strong correlations shown; CHOL, total cholesterol; GLU, fasting glucose; TAG, triglycerides. Strength of the correlation: 0.40–0.59 middle, 0.60–0.79 strong, and 0.80–1.00 very strong.

Abbreviation: HDL, high‐density lipoproteins.

## 3. Discussion

The IGF signaling pathway participates in many vital processes during both intrauterine and early postnatal development but also in adulthood. It controls cellular metabolism; increases mitosis, growth, mobility, and differentiation; and reduces apoptosis [14].

Impaired IGF signaling is associated with pathophysiological conditions such as chronic inflammation, compromised protection against pathogens (viruses), cancer, insulin resistance, dyslipidemia, and cardiovascular diseases [14, 25, 26]. Blocking IGF‐1R can alleviate autoimmune diseases and treat cancer [27–29].

Since there are no reference limits for the levels of any of the growth factors measured, we can only compare the values, not conclude whether they are pathological.

In our study, we documented statistically significant differences in the expression of several representatives of IGF signaling pathways. In a comparison of subjects with and without psoriasis, subjects with psoriasis had lower levels of IGF‐1, IGF‐1R, and IGFBP4 but higher levels of IGFBP3, IGFBP6, and insulin. Differences in correlations between psoriasis subjects and controls were also found. In addition, some IGF family members correlated with metabolic parameters such as total cholesterol, glucose, triglyceride, or HDL. In both groups, IGF‐1 expression correlated with IGF‐R1 and IGF‐2 expression, and IGF‐R1 expression correlated with IGF‐R2 expression. Thus, these results indicate that there is a difference in the expression of members of the IGF signaling pathways between individuals with chronic inflammation and controls, and it is therefore possible that they are involved in the pathogenesis of psoriasis, as will be discussed in the following text.

The ensuing discourse pertains to the results concerning IGFs and their receptors. In the control group, the levels of IGF‐1 and soluble IGF‐1R were higher compared to patients, and the correlation between IGF‐1 and IGF‐R1 was found. IGF‐1 was also correlated with IGF‐2 and IGF‐1R with IGF‐2R in both groups.

We expected that IGF‐1R levels could be higher in the patient group as the cleavage of membrane IGF‐1R is mediated by γ secretase which belongs to intramembrane‐cleaving proteases. Their activity can be enhanced in the presence of cellular stress with increased production of reactive oxygen radicals [30, 31]. IGF‐1 levels were expected to be lower in patients because the expression of IGF‐1 depends on the levels of GH, insulin, TGF‐β, adequate nutrition (especially protein intake), and regular physical activity, and very importantly, it has been described that TNF‐α downregulates the expression of IGF‐1 [32]. Therapy with TNF‐α inhibitors can elevate the levels of IGF‐1 [33]. TGF‐β that has anti‐inflammatory function promotes IGF‐1 production [34]. The levels of TNF‐α are increased in patients with psoriasis compared to control and correlated with disease severity [35, 36]. Psoriasis significantly alters the expression of factors that modulate IGF‐1 expression. IGF‐1/IGF‐1R signalization can drive the inflammatory response. It has been proven that IGF‐1R is expressed in nave CD4+ T cells and influences their polarization into regulatory T cells (Treg) or Th17 cells that are involved in other chronic inflammatory diseases. DiToro et al. [37] showed that Th17 and Treg have upregulated Igf1r, Igfbp4, and Igf2r compared to Th1 and Th2 cells, and IGF‐1R signaling alters the balance between Th17 and Treg subsets in favor of the Th17 subset. The proinflammatory effect of IGF‐1R was also documented in rheumatoid arthritis (RA) where IGF‐1R stimulates the production of proinflammatory cytokine IL‐6 [38]. Inflammatory processes which depend on IGF‐1 signaling can be also driven by induction of pathways associated with aging, especially senescence which is associated with a typical secretory inflammatory phenotype (SASP) [39, 40].

On the other hand, the IGF‐1/IGF‐1R signaling is also responsible for downmodulation of inflammation and maintenance of self‐tolerance and promotes macrophage functional polarization during muscle regeneration from proinflammatory M1 subset to regenerative M2c phenotype [41]. The same effect has on microglia and supports their differentiation to the M2 phenotype, stimulates T regulatory and B cell activity, and maintains self‐tolerance [42, 43]. The results are contradictory, which corresponds to the fact that IGF‐1 can exhibit both proinflammatory and anti‐inflammatory effects.

In our study, it might seem that higher levels of IGF‐1 and IGF‐1R might have proinflammatory potential in controls. However, the fact that soluble IGF‐1Rs block IGF activity must be considered. Lower levels of soluble IGF‐1R in patients may not reflect the membrane expression of IGF‐R1, which may be high, and therefore the binding of IGF‐R1 and IGF‐1, and activity may be higher than in controls. Krane et al. [44] showed that IGF‐1R is upregulated in psoriatic epidermis compared to healthy skin. IGF‐1 is known to not only can promote inflammation but also stimulate keratinocyte and skin fibroblast proliferation and prevent their apoptosis. Its inhibition can reverse skin hyperplasia [23, 45, 46].

Studies that focused on inflammatory and autoimmune diseases have also shown a reduction in IGF‐1. Eivindson et al. [47] included 13 patients with refractory Crohn’s disease and 10 controls in their study. The levels of IGF‐1 and IGFBP3 were lower in patients [47]. Similar conclusions were reached by Katsanos et al. [48] who included 10 patients with Crohn’s disease, 12 with ulcerative colitis, and 30 controls in their study. The levels of IGF‐1 and IGFBP3 were lower in patients compared to controls [48]. On the other hand, Hosback et al. [49] showed that in patients with amyotrophic lateral sclerosis (ALS; 28 patients) or multiple sclerosis (MS; 23 patients). In ALS patients, the levels of IGFs (IGF‐1 + IGF‐2) were higher compared to controls; however, levels of IGFBP1 were lower. In MS patients, the levels of IGF‐1 and IGFBP2 were higher compared to controls [49]. The increase in the levels of IGF‐1 was documented by Tseng et al. [50] in 30 patients with Graves’ disease with hypothyroidism compared to euthyroid controls.

In the context of IGF‐1 levels, the results of fasting insulin and glucose should be mentioned. In our study, patients had higher fasting glucose and insulin levels than controls. The combination of lower IGF‐1 levels with higher glucose and insulin levels may indicate the presence of a metabolic disorder such as insulin resistance [51, 52]. Patients with psoriasis are known to have a higher risk of metabolic syndrome, and insulin resistance is one of the five criteria used to diagnose metabolic syndrome [51].

We did not find differences in the levels of IGF‐2, IGF‐2R, IGFBP1, and IGFBP2, but the correlations between these parameters were found. Correlation differed between the control and patient group. In controls, correlation between IGFBP1 and HDL, IGF‐2R and total cholesterol, IGF‐2R and insulin, IGF‐2R and waist, IGF‐2R and IGFBP2, and IGFBP1 and IGFBP2 were evidenced. In patients, positive IGF‐2R and waist, cholesterol, triglycerides, negative correlation IGFBP6 and glucose, negative IGFBP1 and triglycerides, and positive IGFBP4 and IGF‐1 were found.

The positive correlation between IGFBP1 and HDL in the control group and negative correlation between IGFBP1 and triglycerides may indicate that IGFBP1 has a protective effect in metabolic syndrome. This hypothesis is supported by studies demonstrating reduced IGFBP1 levels in individuals with metabolic syndrome at the risk for cardiovascular disease and diabetes. Gibson et al. [53] documented that lower IGFBP1 levels were associated with low level of HDL, elevated blood pressure, BMI, insulin, etc. Bhangoo et al. [54] proved an association between decreased levels of IGFBP1 and an increase in waist circumferences, triglycerides, BMI, and insulin. Positive correlation was shown between IGFBP1 and adiponectin and HDL [54].

Correlation between IGF‐2R and other factors was found in both the control and the patient group. The most significant was the correlation between IGF‐2R and parameters of metabolic health. IGF‐R2 is activated by IGF‐2, and its main function is to internalize these complexes into lysosomes and to degrade IGF‐2 which is also a ligand for IGF‐1R and insulin receptor. Lee et al. [55] found the correlation between IGF‐2R and BMI and was positively associated with changes in weight. These results correspond with our study; BMI often correlates with waist circumference (in our study, in both groups, Spearman score > 8.1). In controls, IGF‐2R also correlated with IGFBP2. Both factors are associated with lower levels of IGFBP2 and are associated with risk of obesity, type 2 diabetes, or cardiovascular diseases [56]. In the control group, IGFBP2 correlated with IGFBP1, secretion of both is suppressed by insulin, and decrease is associated with obesity [57].

In the patient group, we found a correlation between IGFBP4 and IGF‐1 which stimulate proliferation. IGFBP4 has high affinity to IGF‐1 and blocks its functions. As mentioned previously, the levels of IGF‐1 were lower in patients than in controls, which may indicate that IGF‐1 interacts with IGFBP4, and thus the free plasma fraction of IGF‐1 decreases [57, 58].

We found that levels of three IGFBPs differ between groups. IGFBP4 was higher in the control group, and IGFBP3 and IGFBP6 were higher in the patient group.

IGFBP4 binds to IGF‐1 and IGF‐2 with the same affinity. IGFBP4 also inhibits Wnt/β catenin pathway and thus promotes cardiac stem cell differentiation into cardiomyocytes that support myocardial regeneration and inhibit angiogenesis [59]. The decrease in its expression is associated with different types of tumors and their progression. IGFBP4 has tumor suppressor activity in, for example, hepatocellular cancer, osteosarcoma, or lung cancer [60–62].

The levels of IGFBPs depend not only on their expression but also on their degradation and cleavage. While most other IGFBPs are cleaved by multiple types of proteases, IGFBP4 is relatively resistant to cleavage by the vast majority of proteases and is specifically cleaved by pregnancy‐associated plasma protein A (PAPP‐A). A cleavage of IGFBP4 can occur even in the absence of ligation with IGFs. PAPP‐A activity is higher under inflammatory conditions. Its expression is enhanced by various proinflammatory cytokines, such as IL‐1β and TNF‐α [63]. As we have already stated, psoriasis is associated with higher expression of these proinflammatory cytokines; furthermore, Polat et al. [64] showed that the levels of PAPP‐A are higher in patients with chronic plague psoriasis.

On the other hand, studies conducted in patients with systemic lupus erythematosus (SLE) and its complications demonstrated that IGFBP4 levels are higher compared to controls. Wu et al. [65] suggested that IGFPB4 levels can serve as a marker of lupus nephritis. Similar results were presented by Haque et al. [66] who suggested that IGFBP4 with IGFBP2 and sTNFR2 is a predictive factor of clinical nephritis activity in patients with lupus nephritis.

Lower levels of IGFBP4 and higher levels of its fragments are detected in cardiovascular diseases which are also associated with chronic inflammation and often accompany psoriasis. The presence of fragments is indicative of increased cleavage of IGFBP4 [67]. Mahato et al. [68] measured PAPP‐A levels in patients with acute coronary syndrome together with highly sensitive C‐reactive protein (CRP) and metabolic parameters. PAPP‐A and CRP were higher in patients compared to controls (indicating that there can be an increase in NT‐IGFBP4 as mentioned above). They also described the correlation between PAPP‐A and CRP, apolipoprotein B, insulin, etc. [68]. In our study, insulin was higher in the patient group, and it is known that psoriasis is accompanied by higher levels of CRP [69].

The increased activity of PAPP‐A may explain the reduction in IGFBP4 in psoriasis compared to controls but not in SLE. Here, we could hypothesize that the increase in IGFBP4 levels in SLE is not primarily due to the disease as much as the kidney damage that accompanies the disease. IGFBP4 levels are not increased only in lupus nephritis but also in chronic kidney diseases [70]. However, it is evident that IGFBP4 is involved in numerous processes and is influenced by many factors. Therefore, the results of studies may vary as not all mechanisms of action of IGFPB4 are currently known.

The next measured member of IGFBP is IGFBP3, whose levels were higher in psoriasis patients compared to controls.

IGFBP3 is the most abundant IGFBP that binds to various molecules and receptors; therefore, it has an IGF‐dependent and independent activity. IGFBP3 binds, e.g., transforming growth factor β receptor (TGF‐βR), TGF‐βRV, lactoferrin, plasminogen, fibronectin, heparin, intracellular components, such as retinoid X receptor α (RXR α), nuclear receptor Nur77, EGF receptor (EGFR), etc. IGFBP3 modulates cellular processes such as proliferation, differentiation, apoptosis, autophagy, and DNA damage reparation and plays an important role in angiogenesis, tissue regeneration, or tumorigenesis. The deletion of IGFBP3 is associated with increased metastatic activity in various in vivo cancer models and clinical trials [71, 72].

IGFBP3 is documented to be elevated in the presence of psoriasis. Production is driven by enhanced production of TNF‐α, which level is elevated in psoriasis [32]. Özden et al. [73] compared IGFBP3 expression in subjects with psoriasis and other skin diseases (generalized eczema/nummular dermatitis and pityriasis) and in subjects with psoriasis before and after systemic treatment with cyclosporine or methotrexate. Patients with psoriasis had higher levels of IGFBP3 compared to other skin diseases, and the levels decreased after treatment [73].

The proinflammatory potential of IGFBP3 was described in other studies that evaluated different pathologies. The higher levels of IGFBP3 were detected by Lee et al. [74] in patients with RA compared to patients with osteoarthritis. Matsumoto and Tsurumoto [75] compared levels of IGF‐1 and IGFBP3 between 48 patients with RA and 27 controls. IGF‐1 levels were lower and IGFBP3 higher in RA, similarly to our results [75].

Other autoimmune disease that was studied in the context of IGFBP3 expression is MS. Lanzillo et al. [76] showed that IGFBP3 levels are higher in patients with MS. On the contrary, Akcali et al. [77] found lower levels of IGFBP3 in MS, Eivindson et al. [47] and Katsanos et al. [48] in inflammatory diseases.

Not only autoimmune diseases are associated with elevated levels of IGFBP3. Li et al. [78] showed that hypertrophic cardiomyopathy (myocardium infiltrated with immune cells, increased production of proinflammatory cytokines, cardiac remodeling, and angiogenesis) is associated with increased levels of IGFBP3 compared to controls. They also determined gene expression in H9c2 cells (embryonic rat cardiomyocytes) that overexpress IGFBP3. The expression of genes encoding proteins associated with the extracellular matrix formation (COL1A2, COL3A1; collagen type I α 2/collagen type III α 1 chain; MMP9; matrix metalloproteinase 9) and inflammatory proteins (IL‐6 and TNF‐α) was upregulated [78].

Wang et al. [79] documented that IGFBP3 improved radiosensitivity in oral squamous cancer cells by enhancing the activity of NF‐κB/IL‐6/ROS signaling that induces mitochondrial destruction and apoptosis.

On the other hand, Sakata et al. [80] performed a study on the same type of cancer and showed that IGFBP3 overexpression limited the chemoradiotherapy effectivity. IGFBP3 depletion was accompanied by reduced phosphorylation of DNA–dependent protein kinase catalytic subunit (DNA‐PKcs), which is involved in DNA reparation [80].

Although there are two distinct outcomes, two mechanisms by which IGFBP3 modulates cell activity are also described. It may increase the production of ROS and proinflammatory cytokines and thus induce apoptosis, but it may also increase DNA repair and thus ensure cell survival.

IGFBP3 also induces insulin resistance, which is also associated with metabolic syndrome and psoriasis [81].

The last member of the IGFBP family investigated in our study was IGFBP6. Similarly to IGFBP3, its levels in our study were higher in patients than in controls. IGFBP6 binds IGF and IGF‐2 with a higher affinity for IGF‐2 and engages with multiple membrane, cytoplasmic, and even nuclear targets, as seen with IGFBP3 [29, 82]. Binding to a large number of molecules results in its pleiotropic effects. IGFBP6 is known mainly for its involvement in cancer and modulation of immune system function. Various types of cancer overexpress IGF‐2 which stimulates their proliferation; therefore, IGFBP6 can inhibit their growth, and lower levels are associated with the worst prognosis [83].

Within the immune system, IGFBP6 regulates both innate and acquired immunity; it is necessary for B cell development and T cell migration, stimulation of dendritic cells, neutrophil activation (increased ROS production and myeloperoxidase activity), drives monocyte migration, etc. [84–87]. GFBP6 also regulates aging, specifically cellular senescence. Micutkova et al. found that IGFBP6 delayed replicative senescence, and its downregulation resulted in premature cellular senescence and overexpression prolonged cellular lifespan [88].

The involvement of IGFBP6 in immune responses can be demonstrated by studies regarding its involvement in both protective immune and pathological responses.

Micutkova et al. [89] investigated IGFBP6 levels in patients with HIV and controls. They included monozygotic twins in the study and assessed the genome‐wide DNA methylation pattern and found that IGFBP6 was downregulated [89]. Alunno et al. [90] found that IGFBP6 has a unique function in the development of autoimmune conditions. Their study revealed elevated IGFBP6 levels in individuals with RA compared to those without and additionally demonstrated IGFBP6’s ability to prompt the migration of mononuclear cells in vitro [90]. Wilczak et al. [91] demonstrated that IGFBP6 is overexpressed by oligodendrocytes at the edges of demyelinating plaques, suggesting that it is associated with impaired myelin synthesis and involved in the pathogenesis of MS. Levels were higher in patients with type I diabetes, and the patients with complication have even higher levels of IGFBP6 [92].

IGFBP6 is involved not just in autoimmune inflammation but also in other conditions. Dysregulated expression of IGFBP6 has been observed in atherosclerosis, commonly associated with metabolic disorders like metabolic syndrome, diabetes, dyslipidemia, and obesity. Liu et al. [93] discovered reduced IGFBP6 levels in individuals with unstable carotid plaques, proposing its potential as a predictive biomarker for identifying vulnerable plaques. This effect of IGFBP6 was confirmed by Wang et al. [94] who concluded that the IGFBP6 gene, along with CD68 and PAM, is an effective diagnostic marker of unstable plaques. IGFBP6 is also involved in another complication of metabolic diseases, namely, nonalcoholic fatty liver disease. Stanley et al. showed that IGFBP6 expression was higher with increasing steatosis and was positively associated with nonalcoholic steatohepatitis (NASH) [95]. However, Czogała et al. [95] found that IGFBP6 was lower in obese children and positively correlated with apelin, cholecystokinin, glucagon‐like peptide‐1, and leptin receptor [95]. The study limitations include the relatively low sample size of 34 patients with psoriasis due to strict inclusion criteria and 35 matching controls, geographical localization of Central Europe, solely Caucasian population.

## 4. Conclusions

Psoriasis is a complex disease in which the immune system and metabolic dysregulation play an important role, which was confirmed in our study. Research on the involvement of the IGF‐1 pathway in pathogenesis is very limited. Until now, there have been no studies that have addressed multiple members of the IGF signaling pathway in relation to psoriasis. Mostly single members of this pathway have been investigated and, moreover, often not in the context of psoriasis. In our study, we observed differences in several parameters, IGF‐1, IGF‐1R, and IGFBP4, which were higher in the control group, and IGFBP3, IGFBP6, and insulin, which were higher in the psoriasis group. These findings could reinforce the notion that individuals with psoriasis are susceptible to or exhibit insulin resistance, which is primarily fueled by inflammation. Moreover, insulin resistance appears to exacerbate inflammation, alongside other alterations detected in the expression of the IGF‐1 signaling pathway that we identified. Further studies are encouraged to deepen the understanding of the pathophysiology of psoriasis and its comorbidities in relation to the IGF signaling pathway.

## 5. Material and Methods

### 5.1. Participants

Sixty‐nine people were enrolled in our study over the course of 2021–2023: 34 patients with psoriasis and 35 matching controls. The control group was selected using randomized stratification from the pool of healthy occasional blood donors.

All subjects gave their informed consent for inclusion before they participated in the study. The study was conducted in accordance with the Declaration of Helsinki, and the protocol was approved by the Ethics Committee of the Charles University Hospital in Hradec Kralove, Czech Republic.

The persons with any inflammatory diseases (except for psoriasis in the patient group), pregnancy, and those using nonsteroidal, anti‐inflammatory medications or other chronic medication were excluded. Only patients without any medication for psoriasis in the last 12 months were included in the study. Included patients have never taken any biological therapy medication.

Sixty‐nine participants were enrolled in our study: 35 in the control groups and 34 in the patient group. In the control groups, there were 17 males and 18 females; in the patient group, 17 males and 17 females. The age, BMI, waist circumference, levels of total cholesterol, HDL, and triglycerides did not differ between groups. The only difference was in the levels of fasting glucose which were higher in the patient group (<0.001; Table 3). The blood withdrawals for the examination were taken in the morning while fasting in both groups.

**Table 3:** Demographic and metabolic data of all participants.

C/P	Q1	Q2	Q3	*p*‐value	C/P	Q1	Q2	Q3	*p*‐value
**Age (years)**					**Fasting glucose (mmol/l)**				

C (35)	41.0	49.0	59.0	N	C (35)	4.071	4.61	5.0	<0.001
P (34)	41.8	49.0	62.5		P (26)	4.95	5.4	6.28	

**BMI**					**Total cholesterol (mmol/l)**				

C (34)	24.19	27.4	30.6	N	C (35)	4.42	4.99	5.66	N
P (30)	26.1	29.9	35.0		P (26)	4.36	5.80	5.75	

**Waist circumference (cm)**					**HDL (mmol/l)**				

C (34)	77.8	90.0	104.5	N	C (35)	01.07	1.38	1.98	N
P (30)	86.0	104.0	114.3		P (26)	01.04	1.17	1.56	

	**F**	**M**			**Triglycerides (mmol/l)**				

C	18	17	35	N	C (35)	0.87	1.31	1.87	N
P	17	17	34		P (26)	01.02	1.68	02.02	

*Note:* Q1, Q2, Q3, quartiles; C and P, control and patients; N, nonsignificant difference.

### 5.2. PASI

The severity of psoriasis was scored using a standardized clinical assessment, the Psoriasis Area Severity Index (PASI), which is calculated based on erythema, desquamation, and skin infiltration. PASI of the patients ranged from 11.2 to 40.5 meaning moderate to severe exacerbation of psoriasis in time of admission for the study (median = 15.6; Q1 = 11.2; Q3 = 21.3).

### 5.3. Blood Sample

Blood samples were withdrawn from the cubital vein using BD Vacutainer tubes. Blood serum samples were isolated by centrifugation and stored at −70 °C until analysis. Repeated thawing and freezing were avoided.

### 5.4. Biochemical Parameters

Fasting glycemia, triacylglycerol, cholesterol, HDL, LDL, and non‐HDL were measured in serum at the Institute of Clinical Biochemistry and Diagnostics (University Hospital in Hradec Kralove).

### 5.5. IGF Pathway

To evaluate the levels of members of IGF pathway (IGF‐1, IGF‐1R, IGF‐2, IGF‐2R, IGFBP1, IGFBP2, IGFBP3, IGFBP4, IGFBP6, and insulin), the Quantibody Human IGF Signaling Array (RayBiotech Life Inc., USA; GA) was used according to the manufacturer’s instructions.

### 5.6. Statistics

Statistical evaluation was performed using NCSS 2021 Statistical Software (2021). NCSS, LLC. Kaysville, Utah, USA, ncss.com/software/ncss.

Data are presented by median and interquartile range. Comparisons between patients and controls were performed by two‐sample *t*‐test or nonparametric Mann–Whitney and Kolmogorov–Smirnov tests. Spearman’s rank correlation coefficient was used to assess the association of parameters. The chosen significance level was α = 0.05.

Strength of correlation was assessed using Spearman coefficient, absolute values, and *r*; only moderate and higher strengths were used (>0.4) EVANS, James D., 1996. Straightforward statistics for the behavioral sciences [online]. Pacific Grove: Brooks/Cole Pub. Co [cit. 2017‐05‐17]. ISBN 0534231004 9780534231002.

The estimation of statistical power in this study was based on expert input, taking into account relevant scientific literature, as this is one of the first studies of its kind with limited prior data available for a standard calculation.

## Author Contributions

Borsky Pavel and Borska Lenka conceptualized the study. Krejsek Jan was in control of methodology. Holmannova Drahomira, Borska Lenka, and Fiala Zdenek conducted the investigation. Holmannova Drahomira curated the data. Holmannova Drahomira and Borska Lenka wrote the original draft of the publication. Fiala Zdenek, Krejsek Jan, Hamakova Kvetoslava, Fiala Ondrej, Rehacek Vit, and Maresova Tereza reviewed and edited the manuscript. Cermakova Eva conducted the statistical analyses and visualizations. Borska Lenka and Borsky Pavel supervised the study. Hamakova Kvetoslava and Rehacek Vit administered the project. Borska Lenka and Fiala Zdenek acquired the funding.

## Funding

The study was supported by the Charles University, Faculty of Medicine in Hradec Kralove, the Czech Republic, by project SVV‐2025‐260776. This work was supported by the Cooperatio Program, research area HEAS.

## Disclosure

All authors have read and agreed to the published version of the manuscript.

## Ethics Statement

All subjects signed the informed consent before participating in the study. The study was conducted in accordance with the Declaration of Helsinki, and the protocol was approved by the Ethics Committee of the Faculty Hospital in Hradec Kralove, Czech Republic (Project identification code PROGRES Q40‐09 and Q40‐10, reference number 201705 I83P, date 2 May 2017).

## Consent

The authors have nothing to report.

## Conflicts of Interest

The authors declare no conflicts of interest.

## Supporting Information

Additional supporting information can be found online in the Supporting Information section.

## Supporting information


**Supporting Information** Supporting Information includes Supplementary Table S1 summarizing IGF/IGFBP‐related mRNA tissue/immune‐cell expression patterns and between‐group differences in plasma concentrations in psoriasis patients versus controls.

## Data Availability

The data that support the findings of this study are available upon request from the corresponding author. The data are not publicly available due to privacy or ethical restrictions.
