# Supplementary material for: Differences in the Expression of Insulin‐Like Growth Factor Signaling Pathway Members in Patients With Psoriasis Vulgaris and Controls
Source: Mediators Inflamm. 2026 Feb 2;2026:2136373. doi: 10.1155/mi/2136373 (PMC12862997; doi:10.1155/mi/2136373)
Supplement: Supplementary file 1 — Supporting Information Supporting Information includes Supplementary Table S1 summarizing IGF/IGFBP‐related mRNA tissue/immune‐cell expression patterns and between‐group differences in plasma concentrations in psoriasis patients versus controls. [file MI-2026-2136373-s001.docx]

SUPPLEMENTARY DATA

**Table 1. Measured parameters of mRNA expression and differences in plasma concentration between patients with psoriasis and controls**

|  | mRNA | Immune cells | Patients | Controls |
| --- | --- | --- | --- | --- |
| IGF-1 | all ↓tissue specificity | B cells | ↓ | ↑ |
| IGF2 | all ↑placenta | - | 0 | 0 |
| IGFR1 | all ↓ tissue specificity | all ↑Neu | ↓ | ↑ |
| IGFR2 | all ↑skeletal muscle | all ↑Neu | 0 | 0 |
| IGFBP1 | liver, ovary, placenta, endometrium | - | 0 | 0 |
| IGFBP2 | all ↑pancreas | - | 0 | 0 |
| IGFBP3 | all ↑ liver, placenta | pDC, NK | ↑ | ↓ |
| IGFBP4 | all ↓tissue specificity | NK, MAIT, T and B cells | ↓ | ↑ |
| IGFBP6 | all ↑choroid plexus | Eo, Mo, T cells, mDC | ↑ | ↓ |

The table shows the mRNA expression of the measured parameters in tissues and immune cells and the differences in plasma concentrations of the measured parameters between psoriasis patients and healthy controls in ourstudy.

Some parameters are expressed in all tissues (all) with low tissue specificity, some are expressed in all tissues but have increased expression (↑) in specific tissues, and some are expressed only in specific tissues. The same description of expression applies to immune cells. ↓↑ for patients and controls means that the values were lower or higher in this group when compared with each other. O indicates that there was no difference between the values of the measured parameters between the groups.

Abbreaviations: Eo, eosinophils; MAIT, mucosal-associated invariant T cells; mDC, myeloid Dendritic Cell; Mo, monocytes; Neu, neutrophils; NK, Natural Killer cells; pDC, plasmocytoid Dendritic Cell; proteinatlas.org.
